# The New Antibacterial Properties of the Plants: *Quo vadis* Studies of Anti-virulence Phytochemicals?

**DOI:** 10.3389/fmicb.2021.667126

**Published:** 2021-05-07

**Authors:** José Luis Díaz-Nuñez, Rodolfo García-Contreras, Israel Castillo-Juárez

**Affiliations:** ^1^Laboratorio de Fitoquímica, Posgrado de Botánica, Colegio de Postgraduados, Texcoco, Mexico; ^2^Departamento de Microbiología y Parasitología, Facultad de Medicina, Universidad Nacional Autónoma de México, Ciudad de México, Mexico

**Keywords:** antimicrobial phytochemicals, anti-virulence, quorum sensing, virulence factors, adjuvants

## Abstract

The recent increase in bacterial resistance to antibiotics has motivated the resurgence of the study of natural antimicrobial products. For centuries, plants have been recognized for their bactericidal properties. However, in the last two decades, it has been reported that several plant derived metabolites at growth subinhibitory concentrations also tend to have anti-virulence properties, since they reduce the expression of factors that cause damage and the establishment of pathogenic bacteria. In this area of study, plants have been positioned as one of the main natural sources of anti-virulence molecules, but only a small portion of the plant species that exist have been investigated. Also, anti-virulence studies have been primarily focused on analyzing the ability of extracts and compounds to inhibit quorum sensing and biofilms formation *in vitro*. This mini-review discusses the current panorama, the trends in the study of anti-virulence phytochemicals, as well as their potential for the development of antibacterial therapies.

## Introduction

Bacteria are social cells that use quorum sensing (QS) to communicate with organisms of the same species, between species, as well as with other domains of life (Banerji et al., 2020). QS systems (QSS) involve the release of chemical signals called autoinducers, to perceive the presence and concentration of other cells (Castillo-Juárez et al., 2017). This allows them to exhibit multicellular behaviors and regulate the gene expression of various phenotypes at the population level, as among them, production of metabolites (pigments, antibiotics) and virulence factors, including the formation of biofilms (Castillo-Juárez et al., 2015). It is estimated that 80% of chronic bacterial infections form biofilms that promote adherence to host cells and allow them to withstand massive doses of antibiotics and evade the immune response (Townsley and Shank, 2017).

Anti-virulence activity (anti-pathogenic or anti-infectious) is a broad concept that refers to the ability to prevent production of the factors responsible for establishment, damage and spread, but without affecting bacterial viability (LaSarre and Federle, 2013; Totsika, 2016). It has been proposed that development of anti-virulence therapies is a viable strategy for control of bacterial infections, with the possibility of avoiding or reducing the appearance of resistance (Defoirdt, 2018; Scoffone et al., 2019). In the last two decades, many plant species and phytochemicals have been identified as having anti-QS and anti-biofilm properties (Silva et al., 2016; Muñoz-Cazares et al., 2017). In this mini-review, the current situation of anti-virulence phytochemicals, the evidence, and the challenges faced by this field of research were analyzed.

## Anti-Virulence Properties of Bactericidal Phytochemicals

**FIGURE 1:**
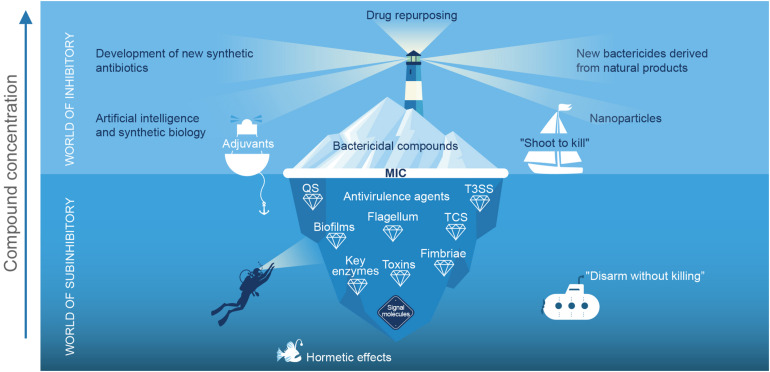
Schematic representation of the current status of antimicrobial strategies: “The world of the inhibitory”. The tip of the iceberg represents the bactericidal compounds that have been discovered, while the light of the beacon searches for current strategies to prevent the “dusk” of this class of molecules. The resurgence of research in natural products, repurposing drugs for use as antibiotics, nanoparticles, chemical synthesis of new bactericides, as well as advances in computer science, omics, artificial intelligence, and synthetic biology are playing a relevant role in the development of new bactericidal compounds (Zakeri and Lu, 2013; Pushpakom et al., 2018; Li et al., 2019; Stokes et al., 2020). However, in this analogy the strategy of “shooting to kill,” allows some pathogenic microorganisms to live and generate resistance; in addition, in the “crossfire” beneficial microorganisms are eliminated. In the “world of the sub-inhibitor,” the number of bioactive molecules to be explored is greater, and the strategy is based on “disarming without killing,” in theory, will not induce resistance. At values below the minimum inhibitory concentration (MIC), the compounds exhibit different effects, among which are anti-virulence, and signal molecule activity, and they have hormetic and adjuvant effects (Cox et al., 2017). The term “anti-virulence agent” also includes peptides, enzymes, and antibodies. QS, quorum sensing; TS33, type 3 secretion system and TCS, two-component systems.

Natural products of microbial origin are the main source of bactericidal compounds, which had a “golden age” in the middle of the last century and prompted the development of commercial antibiotics (Brown and Wright, 2016). However, despite being one of humanity’s greatest scientific discoveries, the alarming increase in bacterial resistance has put their efficacy and future use at risk (López-Jácome et al., 2019). Nevertheless, it should be noted that only a small proportion of the total bioactive molecules in nature have been explored, so new antibiotics continue to be sought (Li et al., 2019; Stokes et al., 2020). Different strategies are being used to avoid the “nightfall” of this class of molecules and favor the emergence of a second “golden age” (Figure 1).

Although the trend in development of antimicrobials has focused on their growth inhibitory properties, it has also been reported that antibiotics at sub-inhibitory concentrations can modulate QSS, virulence (Davies et al., 2006; Khan et al., 2020b), and biofilm formation (Khan et al., 2020a). For example, linezolid has been reported to reduce production of virulence factors from *Staphylococcus aureus* (Bernardo et al., 2004). Also, azithromycin interferes with QS, reducing gene expression and the production of autoinducers in *Pseudomonas aeruginosa*, while streptomycin does so in *Acinetobacter baumannii* (Nalca et al., 2006; Saroj and Rather, 2013). Interestingly, this phenomenon has also been identified in drugs of mass consumption such as aspirin (El-Mowafy et al., 2014) and ibuprofen (Dai et al., 2019), in fermented products, and in various bactericidal phytochemicals (Muñoz-Cazares et al., 2017). Thus, the effect of metabolites at low concentrations on microbial social networks and virulence regulation is a frontier issue that increases the number of molecules to be explored at sub-inhibitory concentrations (Figure 1).

## Challenges and Trends in the Study of Anti-Virulence Phytochemicals

In recent decades, it has been reported that many natural products, especially phytochemicals, exhibit anti-virulence properties when evaluated at subinhibitory concentrations (Brown and Wright, 2016; Silva et al., 2016; Muñoz-Cazares et al., 2017; Mulat et al., 2019). Within natural products, plants are an important source of anti-virulence molecules, but most have been evaluated only *in vitro*. They are not new chemical structures, and many have been reported as bactericidal (Muñoz-Cazares et al., 2017).

The trend in studies related to identification of the anti-virulence mechanism of phytochemicals has focused on showing that they interrupt some element of the QSS. The *in silico* approach has been widely used through computational methods, such as molecular docking, to suggest the interaction of phytochemicals with LuxR-type receptor proteins and/or LuxI-type synthases (Deryabin et al., 2019). Multi-omics analysis (proteomic, transcriptomic, and metabolomic) has shown that some phytochemicals interfere with the expression of various QS genes, but also with other non-QS genes. Such is the case of coumarin, which reduces the expression of genes involved in QS, type 3 secretion system (T3SS), and metabolism of cyclic diguanylate in *P. aeruginosa* (Zhang et al., 2018). In the same way, ajoene reduces the expression of virulence factors in *P. aeruginosa* and *S. aureus* by inhibiting small regulatory RNAs (Jakobsen et al., 2017; Table 1). However, some reports identify natural products that can inhibit other anti-virulence targets such as other secretion systems, adhesion molecules, toxins, two-component systems, key enzymes, curli, flagellum as well as metabolic processes involved in the formation and maturation of biofilms (Muñoz-Cazares et al., 2018).

**TABLE 1:** Main antibacterial effects of phytochemicals at sub-inhibitory concentrations.

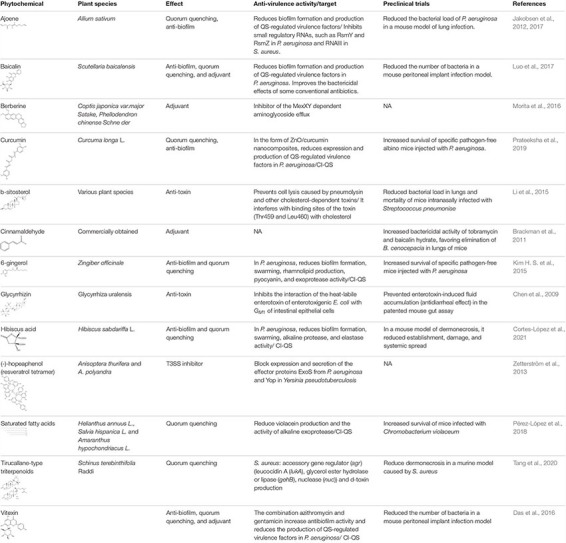

*T3SS, type 3 secretion system; NA, not available; CI-QS, possible competitive inhibition with QS receptor proteins.*

Several anti-virulence phytochemicals have been shown to reduce establishment and damage caused by bacteria *in vivo*, mainly in the nematode *Caenorhabditis elegans* in murine models (Castillo-Juárez et al., 2015) and animals of importance in aquaculture (Zhao et al., 2015). Also, they have preventive effects on phytopathogenic bacterial infections in some models with *Arabidopsis thaliana*, *Brassica oleracea* and *Solanum tuberosum*, among others (Jhosi et al., 2015; Sivaranjani et al., 2016). Although there is evidence that disruption of virulence by phytochemicals is a potential strategy to prevent disease, there are emerging issues and challenges that have been little studied and are detailed below (Mulat et al., 2019).

### Anti-virulence Phytochemicals and Their Role in the Daily Diet

One of the trends is related to the role of anti-virulence phytochemicals present in edible plant species and their ability to prevent infectious processes (Givskov, 2012; McCarthy and O’Gara, 2015). Although it is thought that plants are unlikely to contain concentrations of phytochemicals high enough to counteract established bacterial infections, it has been proposed that their continuous consumption may prevent development of chronic infections (Givskov, 2012). This is still difficult to conclude, but, QS inhibitors have been identified in some edible species such as garlic (Bjarnsholt et al., 2005), oilseeds (Pérez-López et al., 2018), and hibiscus acid isolated from *Hibiscus sabdariffa* (Cortes-López et al., 2021), which have been shown to have anti-virulence properties and reduce bacterial pathogenicity in mice (Table 1).

### Phytochemicals as Inducers de QS

Bactericidal molecules commonly have a dose-response effect, but at subinhibitory concentrations, they can exhibit multiple effects on bacterial cells (Davies et al., 2006; Figure 1). Hormesis is a phenomenon that commonly occurs at low concentrations and is characterized by antagonistic activities (stimulate/inhibit) exhibited by the same molecule, depending on the concentration (Mattson, 2008; Martel et al., 2019; Figure 1). Although the hormetic effect of phytochemicals has been reported in other biological activities (Martel et al., 2019), their clinical use could be complicated by a change in concentration that can stimulate virulence. It has been reported that furanone and other inhibitors can inhibit or activate QS depending on the concentration (Martinelli et al., 2004; Welsh et al., 2015; Yao et al., 2019). Similarly, some natural products with no bactericidal activity can stimulate the formation of biofilms (Ranieri et al., 2018). In the case of phytochemicals, the hormetic effects have been little studied, but linalool and eugenol have been reported to have this type of effect on biofilm formation and the rhamnolipids production of *P. aeruginosa* PAO1 (Kim Y. G. et al., 2015). Also, coumarin was reported to affect swarming of *Ralstonia solanacearum* (Chen et al., 2016) and capsaicin to affect biofilm formation in *P. aeruginosa* PAO1 and *Serratia marcescens* (Rivera et al., 2019).

### Effects of Anti-virulence Phytochemicals on the Microbiome

So far, inhibition of virulence regulation systems appears to be advantageous in combating pathogenic bacteria. However, there are still few studies on its effect on the QS systems of beneficial bacteria, the microbiome in general, or on the host (McCarthy and O’Gara, 2015; Lakes et al., 2020). Unlike *in vitro* monoculture trials, pathogenic bacteria develop in polymicrobial communities where they interact with environmental factors and different specific signaling molecules (many of them still unknown) that can determine the virulence of the pathogen (Banerji et al., 2020). We now know that the intestinal microbiome participates in many aspects of health; microbe-host interactions influence obesity, inflammatory and digestive processes, and certain psychiatric conditions, among others (Burdet et al., 2019). In this context, it has been seen that alteration of the microbiome by exposure to penicillin at sublethal doses in the early stages of development induces metabolic alterations and affects expression of genes involved in host immunity, favoring obesity induced by a high-fat diet (Cox et al., 2014). Similarly, some phytochemicals commonly ingested in the diet (phenolic compounds, terpenes, and alkaloids) affect intestinal bacterial groups and it is suggested that they may affect host microbial ecology and physiology when administered at bactericidal concentrations (Lakes et al., 2020). Morever, recent studies suggest that in complex microbial communities, interference with QS severely affects microbiome composition. However, up to the moment of this review, we did not find reports related to the effect of anti-virulence phytochemicals on the microbiome at sub-inhibitory concentrations (Nguyen et al., 2019; Waheed et al., 2020).

### Development of Combination Anti-virulence Therapies

Some of the strategies to potentiate the efficacy of anti-virulence molecules are the development of combination therapies of inhibitors with different targets (Fong et al., 2018; Ranieri et al., 2018). However, although the mechanism of action of most phytochemicals is unknown, it is highly feasible that they act at various sites to reduce virulence, as some transcriptomic and proteomic studies have revealed (Jakobsen et al., 2017; Zhang et al., 2018).

Adjuvant activity is a property that has been recently identified in several natural anti-virulence products and that helps to restore the activity of antibiotics against sensitive and resistant strains (Cox et al., 2017). Such are the cases of baicalin (Luo et al., 2017), berberine (Morita et al., 2016), cinnamaldehyde (Brackman et al., 2011), and vitexin (Das et al., 2016; Table 1). Although the mechanisms involved in this phenomenon are not known, in the case of berberine it has been reported that it restores the bactericidal activity of aminoglycosides because it blocks the efflux pumps that expel these antibiotics (Morita et al., 2016).

### Induction of Resistance

The premise of the anti-virulence strategy is based on removing the pathogenicity of microorganisms without directly affecting their viability, so that, arguably, strong selection pressures are not generated to induce resistance (McCarthy and O’Gara, 2015). However, some reports indicate that furanone C-30 at subinhibitory concentrations generates resistance by a mechanism that involves the expression of expulsion pumps for this compound (Maeda et al., 2012; García-Contreras et al., 2016). Also, it is suggested that the presence of “cheaters” (bacteria that do not participate in collective communication but do benefit from the products that are produced) in populations may favor resistance because they would be naturally resistant to QS inhibitors (Kalia et al., 2014). Moreover, a recent finding in *Escherichia coli* suggest that QS inhibition may promote conjugation of plasmids and increase the mutation rate, hence favoring the generation of resistance (Li et al., 2021). This is one of the most debated issues in this area; however, to date no reports have shown that anti-virulence phytochemicals induce resistance.

### Patents, Preclinical, and Clinical Studies

Although several patents for anti-virulence agents have been published, most focus on their ability to block QS or prevent biofilm formation, and there are few studies that corroborate the effect at the preclinical (Table 1) or clinical level (Kalia et al., 2019). In the specific case of phytochemicals, studies on their ability to act on biofilms abound, but clinical trials remain scarce (Reuter et al., 2016). In this regard, the study of garlic as an anti-QS agent in the treatment of cystic fibrosis stands out; the study reports a reduction in symptoms and an improvement in lung function (Smyth et al., 2010). Another is the anti-biofilm formulation based on *Hymus vulgaris*, *Eugenia caryophyllus*, and lactobacilli for the treatment of bacterial vaginosis, in which administration by slow-release capsules was able to reduce signs and symptoms in 80% of patients (Murina et al., 2018).

## Conclusion and Perspectives

Among natural products, plants have played a discrete role in the discovery of bactericidal compounds, but they have thus far been positioned themselves as the main source of anti-virulence molecules. However, studies of anti-virulence phytochemicals have focused mainly on analyzing their quorum quenching and antibiofilm properties *in vitro*. The few preclinical trials conducted have identified only preventive effects and they have not yet been shown to counteract established infections. In this regard, it is suggested that the anti-virulence activity registered in bacterial monocultures and ideal growth conditions (rich media) cannot always be extrapolated to the complex conditions that occur in the host (Davies et al., 2006; Turovskiy et al., 2007; Juárez-Rodríguez et al., 2021). Reports exist that indicate that host environmental factors and the presence of other microbial species may interfere with virulence expression (Sabag-Daigle et al., 2012; Ismail et al., 2016). Recently, it was reported that myristic acid, which reduces virulence *in vitro*, behaves as a signal molecule stimulating the pathogenicity of *P. aeruginosa* in a dermonecrotic mouse model (Juárez-Rodríguez et al., 2021). Furthermore, it has been discovered that in some murine models the T3SS are the main virulence determinants, while the QSS seems to have a more discrete role (Miki et al., 2010; Soto-Aceves et al., 2019; Juárez-Rodríguez et al., 2021). Thus, deciphering the ecological context in which virulence is regulated *in vivo* will be decisive for the development of effective therapies.

On the other hand, some required characteristics of an ideal anti-virulence molecule have been proposed. Most of them are the same as those expected for other bioactive compounds: high specificity, stability and absence of side effects (Kalia et al., 2019). However, other desirable properties such as not generating resistance or not negatively altering the host microbiome, have been little studied. Another important characteristic is that they should have no bactericidal activity against the pathogen or the microbiome (Davies et al., 2006). Also, hormetic effects that can stimulate virulence should be absent, and they should have the ability to inhibit several anti-virulence targets simultaneously. The latter can help reduce possible side effects derived from the administration of multi-drug therapies and decrease resistance selection.

Furthermore, it is important to expand research into other anti-virulence targets on which the phytochemicals may be acting. One of them is the T3SS, which even though various synthetic molecules have been described that inhibit it, the number of phytochemicals reported with this activity is scarce. In this regard, the preclinical results obtained with (-)-hopeaphenol are very important (Zetterström et al., 2013; Table 1). Also, anti-toxin properties are important, as in the case of β-sitosterol and glycyrrhizin, which protect from damage caused by bacterial toxins (Chen et al., 2009; Li et al., 2015; Table 1). Finally, the use of nanoparticles to potentiate the effect of phytochemicals is a strategy with which good results have been obtained at the preclinical level, as has been demonstrated with curcumin (Prateeksha et al., 2019; Table 1). All these trends contribute to the resurgence of the study of natural antibacterial products, with great potential to help solve the current crisis of antibiotics.

## Author Contributions

All the authors have contributed equally to the proposal, writing, and editing of the manuscript and also read and approved the final version of the manuscript.

## Conflict of Interest

The authors declare that the research was conducted in the absence of any commercial or financial relationships that could be construed as a potential conflict of interest.

## References

[B1] Banerji R. Kanojiya P. Saroj S. D. (2020). Role of interspecies bacterial communication in the virulence of pathogenic bacteria. *Crit. Rev. Micro.* 46 136–146. 10.1080/1040841X.2020.1735991 32141353

[B2] Bernardo K. Pakulat N. Fleer S. Schnait A. Utermöhlen O. Krut O. (2004). Subinhibitory concentrations of linezolid reduce *Staphylococcus aureus* virulence factor expression. *Antimicrob. Agents. Chemother.* 48 546–555. 10.1128/AAC.48.2.546-555.2004 14742208 PMC321544

[B3] Bjarnsholt T. Jensen P. Ø Rasmussen T. B. Christophersen L. Calun H. Hentzer M. (2005). Garlic blocks quorum sensing and promotes rapid clearing of pulmonary *Pseudomonas aeruginosa* infections. *Microbiology* 151 3873–3880. 10.1099/mic.0.27955-0 16339933

[B4] Brackman G. Cos P. Maes L. Nelis H. J. Coenye T. (2011). Quorum sensing inhibitors Increase the susceptibility of bacterial biofilms to antibiotics *in vitro* and *in vivo*. *Antimicrob. Agents. Chemother.* 55 2655–2661. 10.1128/AAC.00045-11 21422204 PMC3101409

[B5] Brown E. D. Wright G. D. (2016). Antibacterial drug discovery in the resistance era. *Nature* 529 336–343. 10.1038/nature17042 26791724

[B6] Burdet C. Nguyen T. T. Duval X. Ferreira S. Andremont A. Guedj J. (2019). Impact of antibiotic gut exposure on the temporal changes in microbiome diversity. *Antimicrob. Agents. Chemother.* 63:e00820-19. 10.1128/AAC.00820-19 31307985 PMC6761552

[B7] Castillo-Juárez I. López-Jácome L. E. Soberón-Chávez G. Tomás M. Lee J. Castaneda-Tamez P. (2017). Exploiting quorum sensing inhibition for the control of *Pseudomonas aeruginosa* and *Acinetobacter Baumannii* biofilms. *Curr. Top. Med. Chem.* 17 1915–1927. 10.2174/1568026617666170105144104 28056745

[B8] Castillo-Juárez I. Maeda T. Mandujano-Tinoco E. A. Tomás M. Pérez-Eretza B. García-Contreras S. J. (2015). Role of quorum sensing in bacterial infections. *World J. Clin. Cases* 3 575–598. 10.12998/wjcc.v3.i7.575 26244150 PMC4517333

[B9] Chen J. C. Ho T. H. Chang Y. S. Wu S. L. Li C. C. Hsiang C. Y. (2009). Identification of *Escherichia coli* enterotoxin inhibitors from traditional medical herbs *in silico*, *in vitro* and *in vivo* analyses. *J. Ethnopharmacol.* 121 372–378. 10.1016/j.jep.2008.11.011 19063958

[B10] Chen J. N. Yu Y. M. Li S. L. Ding W. (2016). . Resveratrol and coumarin: novel agricultural antibacterial agent against *Ralstonia solanacearum in vitro* and *in vivo*. *Molecules* 21 1–18. 10.3390/molecules21111501 27834875 PMC6273507

[B11] Cortes-López H. Castro-Rosas J. García-Contreras R. Rodríguez-Zavala J. S. Díaz-Guerrero M. González-Pedrajo B. (2021). Anti-virulence activity of a dietary phytochemical: hibiscus acid isolated from *Hibiscus sabdariffa* reduces the virulence of *Pseudomonas aeruginosa* in a mouse infection model. *J. Med. Food* 1557–7600. 10.1089/jmf.2020.0135 [Epub ahead of print]. 33751918

[B12] Cox G. Sieron A. King A. M. De Pascale G. Pawlowski A. C. Koteva K. (2017). A common platform for antibiotic dereplication and adjuvant discovery. *Cell Chem. Biol.* 24 98–109. 10.1016/j.chembiol.2016.11.011 28017602

[B13] Cox L. M. Yamanishi S. Sohn J. Alekseyenko A. V. Leung J. M. Cho I. (2014). Altering the intestinal microbiota during a critical developmental window has lasting metabolic consequences. *Cell* 158 705–721. 10.1016/j.cell.2014.05.052 25126780 PMC4134513

[B14] Dai L. Wu T. Xiong Y. Ni H. Ding Y. Zhang W. (2019). Ibuprofen-mediated potential inhibition of biofilm development and quorum sensing in *Pseudomonas aeruginosa*. *Life Sci.* 237 1–9. 10.1016/j.lfs.2019.116947 31605708

[B15] Das M. C. Sandhu P. Gupta P. Rudrapaul P. De U. C. Tribedi P. (2016). Attenuation of *Pseudomonas aeruginosa* biofilm formation by Vitexin: a combinatorial study with azithromycin and gentamicin. *Sci. Rep.* 6:23347. 10.1038/srep23347 27000525 PMC4802347

[B16] Davies J. Spiegelman G. B. Yim G. (2006). The world of subinhibitory antibiotic concentrations. *Curr. Opin. Microbiol.* 9 445–453. 10.1016/j.mib.2006.08.006 16942902

[B17] Defoirdt T. (2018). Quorum-sensing systems as targets for antivirulence therapy. *Trends Microbiol.* 26 313–328. 10.1016/j.tim.2017.10.005 29132819

[B18] Deryabin D. Galadzhieva A. Kosyan D. Duskaev G. (2019). Plant-derived inhibitors of AHL-mediated quorum sensing in bacteria: modes of action. *Int. J. Mol. Sci.* 20 1–22. 10.3390/ijms20225588 31717364 PMC6888686

[B19] El-Mowafy S. A. Abd El Galil K. H. El-Messery S. M. Shaaban M. I. (2014). Aspirin is an efficient inhibitor of quorum sensing, virulence and toxins in *Pseudomonas aeruginosa*. *Microb Pathog.* 74 25–32. 10.1016/j.micpath.2014.07.008 25088031

[B20] Fong J. Zhang C. Yang R. Boo Z. Z. Tan S. K. Nielsen T. E. (2018). Combination therapy strategy of quorum quenching enzyme and quorum sensing inhibitor in suppressing multiple quorum sensing pathways of *P. aeruginosa*. *Sci. Rep.* 8 1–11. 10.1038/s41598-018-19504-w 29348452 PMC5773576

[B21] García-Contreras R. Maeda T. Wood T. K. (2016). Can resistance against quorum-sensing interference be selected? *ISME J.* 10 4–10. 10.1038/ismej.2015.84 26023871 PMC4681860

[B22] Givskov M. (2012). Beyond nutrition: health-promoting foods by quorum-sensing inhibition. *Future Microbiol.* 7 1025–1028. 10.2217/fmb.12.84 22953702

[B23] Ismail A. S. Valastyan J. S. Bassler B. L. (2016). A host-produced autoinducer-2 mimic activates bacterial quorum sensing. *Cell Host Microbe* 19 470–480. 10.1016/j.chom.2016.02.020 26996306 PMC4869860

[B24] Jakobsen T. H. van Gennip M. Phipps R. K. Shanmugham M. S. Christensen L. D. Alhede M. (2012). Ajoene, a sulfur-rich molecule from garlic, inhibits genes controlled by quorum sensing. *Antimicrob. Agents Chemother.* 56 2314–2325. 10.1128/AAC.05919-11 22314537 PMC3346669

[B25] Jakobsen T. H. Warming A. N. Vejborg R. M. Moscoso J. A. Stegger M. Lorenzen F. (2017). A broad range quorum sensing inhibitor working through sRNA inhibition. *Sci. Rep.* 7 1–10. 10.1038/s41598-017-09886-8 28851971 PMC5575346

[B26] Jhosi J. R. Burdman S. Lipsky A. Yedidia I. (2015). Effects of plant antimicrobial phenolic compounds on virulence of the genus *Pectobacterium*. *Res. Microbiol.* 166 535–545. 10.1016/j.resmic.2015.04.004 25981538

[B27] Juárez-Rodríguez M. M. Cortes-López H. García-Contreras R. González-Pedrajo B. Díaz-Guerrero M. Martínez-Vázquez M. (2021). Dodecanoic and tetradecanoic acids with in vitro anti-virulence properties increase the pathogenicity of *Pseudomonas aeruginosa* in a murine cutaneous infection model. *Front. Cell. Infect. Microbiol.* 10:597517. 10.3389/fcimb.2020.597517 33585272 PMC7876447

[B28] Kalia V. C. Patel S. K. Kang Y. C. Lee J.-K. (2019). Quorum sensing inhibitors as antipathogens: biotechnological applications. *Biotechnol. Adv.* 37 68–90. 10.1016/j.biotechadv.2018.11.006 30471318

[B29] Kalia V. C. Wood T. K. Kumar P. (2014). Evolution of resistance to quorum sensing inhibitors. *Microb. Ecol.* 68 13–23. 10.1007/s00248-013-0316-y 24194099 PMC4012018

[B30] Khan F. Pham T. N. D. Kim Y. M. (2020a). Alternative strategies for the application of aminoglycoside antibiotics against the biofilm-forming human pathogenic bacteria. *Appl. Microbiol. Biotechnol.* 104 1955–1976. 10.1007/s00253-020-10360-1 31970432

[B31] Khan F. Pham T. N. D. Oloketuyi F. S. Kim Y. M. (2020b). Antibiotics and their different application strategies in controlling the biofilm forming pathogenic bacteria. *Curr. Pharmaceut. Biotechnol.* 2 270–286. 10.2174/1389201020666191112155905 31721708

[B32] Kim H. S. Lee S. H. Byun Y. Park H. D. (2015). 6-Gingerol reduces *Pseudomonas aeruginosa* biofilm formation and virulence via quorum sensing inhibition. *Sci. Rep.* 5:8656. 10.1038/srep08656 25728862 PMC4345325

[B33] Kim Y. G. Lee J. H. Kim S. I. Baek K. H. Lee J. (2015). Cinnamon bark oil and its components inhibit biofilm formation and toxin production. *Int. J. Food Microbiol.* 195 30–39. 10.1016/j.ijfoodmicro.2014.11.028 25500277

[B34] Lakes J. E. Richards C. I. Flythe M. D. (2020). Inhibition of Bacteroidetes and Firmicutes by select phytochemicals. *Anaerobe* 61 1–6. 10.1016/j.anaerobe.2019.102145 31918362 PMC7441489

[B35] LaSarre B. Federle M. J. (2013). Exploiting quorum sensing to confuse bacterial pathogens. *Microbiol. Mol. Biol. Rev.* 77 73–111. 10.1128/MMBR.00046-12 23471618 PMC3591984

[B36] Li F. Wang Y. Li D. Chen Y. Dou Q. P. (2019). Are we seeing a resurgence in the use of natural products for new drug discovery? *Expert Opin. Drug Discov.* 14 417–420. 10.1080/17460441.2019.1582639 30810395

[B37] Li H. Zhao X. Wang J. Dong Y. Meng S. Li R. (2015). Beta-sitosterol interacts with pneumolysin to prevent *Streptococcus pneumoniae* infection. *Sci. Rep.* 5:17668. 10.1038/srep17668 26631364 PMC4668377

[B38] Li X. Lui Y. Wang Y. Lin Z. Wang D. Sun H. (2021). Resistance risk induced by quorum sensing inhibitors and their combined use with antibiotics: mechanism and its relationship withtoxicity. *Chemosphere* 265:129153. 10.1016/j.chemosphere.2020.129153 33302207

[B39] López-Jácome E. Franco-Cendejas R. Quezada H. Morales-Espinosa R. Castillo-Juárez I. González-Pedrajo B. (2019). The race between drug introduction and appearance of microbial resistance. *Curr. Opin. Pharmacol.* 48 48–56. 10.1016/j.coph.2019.04.016 31136908

[B40] Luo J. Dong B. Wang K. Cai S. Liu T. Cheng X. (2017). Baicalin inhibits biofilm formation, attenuates the quorum sensing-controlled virulence and enhances *Pseudomonas aeruginosa* clearance in a mouse peritoneal implant infection model. *PLoS One* 12:e0176883. 10.1371/journal.pone.0176883 28453568 PMC5409170

[B41] Maeda T. García-Contreras R. Pu M. Sheng L. García L. R. Tomás M. (2012). Quorum quenching quandary: resistance to antivirulence compounds. *ISME J.* 6 493–501. 21918575 10.1038/ismej.2011.122 PMC3280137

[B42] Martel J. Ojcius D. M. Ko Y.-F. Ke P.-Y. Wu C.-Y. Peng H. H. (2019). Hormetic effects of phytochemicals on health and longevity. *Trends Endocrinol. Metab.* 30 335–345. 10.1016/j.tem.2019.04.001 31060881

[B43] Martinelli D. Grossmann G. Séquin U. Brandl H. Bachofen R. (2004). Effects of natural and chemically synthesized furanones on quorum sensing in *Chromobacterium violaceum*. *BMC Microbiol.* 4:25. 10.1186/1471-2180-4-25 15233843 PMC509243

[B44] Mattson M. P. (2008). Hormesis defined. *Ageing Res. Rev.* 7 1–7. 10.1016/j.arr.2007.08.007 18162444 PMC2248601

[B45] McCarthy R. R. O’Gara F. (2015). The impact of phytochemicals present in the diet on microbial signalling in the human gut. *J. Funct.* 14 684–691. 10.1016/j.jff.2015.02.032

[B46] Miki T. Iguchi M. Akiba K. Hosono M. Sobue T. Danbara H. (2010). *Chromobacterium* pathogenicity island 1 type III secretion system is a major virulence determinant for *Chromobacterium violaceum*-induced cell death in hepatocytes. *Mol. Microbiol.* 77 855–872. 10.1111/j.1365-2958.2010.07248.x 20545857

[B47] Morita Y. Nakashima K. Nishino K. Kotani K. Tomida J. Inoue M. (2016). Berberine is a novel type efflux inhibitor which attenuates the MexXY-mediated aminoglycoside resistance in *Pseudomonas aeruginosa*. *Front. Microbiol.* 7:1223. 10.3389/fmicb.2016.01223 27547203 PMC4975076

[B48] Mulat M. Pandita A. Khan F. (2019). Medicinal plant compounds for combating the multi-drug resistant pathogenic bacteria: a review. *Curr. Pharm. Biotechnol.* 20 183–196. 10.2174/1872210513666190308133429 30854956

[B49] Muñoz-Cazares N. García-Contreras R. Soto-Hernández M. Martínez-Vázquez M. Castillo-Juárez I. (2018). “Chapter 10: Natural products with quorum quenching independent anti-virulence properties,” *Studies in Natural Products Chemistry (Bioactive Natural Products)*, Vol. 57 (Amsterdam: Elsevier Science Publishers), 327–351. 10.1016/B978-0-444-64057-4.00010-7

[B50] Muñoz-Cazares N. García-Contreras R. Pérez-López M. Castillo-Juárez I. (2017). “Phenolic compounds with anti-virulence properties,” in *Phenolic Compounds-Biological Activity*, eds Soto-Hernández M. Tenango M. P. García-Mateos R. (London: IntechOpen), 139–167. 10.5772/66367

[B51] Murina F. Vicarrioto F. Di Francesco S. (2018). Thymol, eugenol and lactobacilli in a medical device for the treatment of bacterial vaginosis and vulvovaginal candidiasis. *New Microbiol.* 41 220–224. 29874389

[B52] Nalca Y. Jhäsch L. Brendenbruch F. Geffers R. Buer J. Häussler S. (2006). Quorum-sensing antagonistic activities of azithromycin in *Pseudomonas aeruginosa* PAO1: a global approach. *Antimicrob. Agents. Chemother.* 50 1680–1688. 10.1128/AAC.50.5.1680-1688.2006 16641435 PMC1472232

[B53] Nguyen P. D. T. Mustapha N. A. Kadokami K. García-Contreras R. Wood T. K. Maeda T. (2019). Quorum sensing between Gram-negative bacteria responsible for methane production in a complex waste sewage sludge consortium. *Appl. Microbiol. Biotechnol.* 103 1485–1495. 10.1007/s00253-018-9553-9 30554390

[B54] Pérez-López M. García-Contreras R. Soto-Hernández M. Rodríguez-Zavala J. Martínez-Vázquez M. Prado-Galbarro F. J. (2018). Anti-quorum sensing activity of seed oils from oleaginous plants and protective effect during challenge with *Chromobacterium violaceum*. *J. Med. Food* 21 356–363. 10.1089/jmf.2017.0080 29172966

[B55] Prateeksha V. R. C. Das K. A. Barik K. S. Singh N. B. (2019). ZnO/Curcumin nanocomposites for enhanced inhibition of *Pseudomonas aeruginosa* virulence via LasR-RhlR quorum sensing systems. *Mol. Pharm.* 16 3399–3413. 10.1021/acs.molpharmaceut.9b00179 31260316

[B56] Pushpakom S. Iorio F. Eyers P. A. Escott K. J. Hopper S. Wells A. (2018). Drug repurposing: progress, challenges and recommendations. *Nat. Rev. Drug Discov.* 18 41–58. 10.1038/nrd.2018.168 30310233

[B57] Ranieri M. R. Whitchurch B. C. Burrows L. L. (2018). Mechanisms of biofilm stimulation by subinhibitory concentrations of antimicrobials. *Curr. Opin. Microbiol.* 45 164–169. 10.1016/j.mib.2018.07.006 30053750

[B58] Reuter K. Steinbach A. Helms V. (2016). Interfering with bacterial quorum sensing. *Perspect. Medicin. Chem.* 8 1–15. 10.4137/PMc.s13209 26819549 PMC4718088

[B59] Rivera M. L. C. Hassimotto N. M. A. Bueris V. Sircili M. P. Almeida F. A. Pinto U. M. (2019). Effect of *Capsicum frutescens* extract, capsaicin, and luteolin on quorum sensing regulated phenotypes. *J. Food Sci.* 84 1477–1486. 10.1111/1750-3841.14648 31132155

[B60] Sabag-Daigle A. Soares J. A. Smith J. N. Elmasry M. E. Ahmer B. (2012). The acyl homoserine lactone receptor, SdiA, of *Escherichia coli* and *Salmonella enterica* serovar Typhimurium does not respond to indole. *Appl. Environ. Microbiol.* 78 5424–5431. 10.1128/AEM.00046-12 22610437 PMC3416396

[B61] Saroj S. D. Rather P. N. (2013). Streptomycin inhibits quorum sensing in *Acinetobacter baumannii*. *Antimicrob. Agents Chemother.* 57 1926–1929. 10.1128/AAC.02161-12 23318804 PMC3623334

[B62] Scoffone V. C. Trespidi G. Chiarelli L. R. Barbieri G. Buroni S. (2019). Quorum sensing as antivirulence target in cystic fibrosis pathogens. *Int. J. Mol. Sci.* 20 1–38. 10.3390/ijms20081838 31013936 PMC6515091

[B63] Silva L. N. Zimmer K. R. Macedo A. J. Trentin D. S. (2016). Plant natural products targeting bacterial virulence factors. *Chem. Rev.* 116 9162–9236. 10.1021/acs.chemrev.6b00184 27437994

[B64] Sivaranjani M. Krishnan S. R. Kannappan A. Ramesh M. Ravi A. V. (2016). Curcumin from *Curcuma longa* affects the virulence of *Pectobacterium wasabiae* and *P. carotovorum* subsp. *carotovorum* via quorum sensing regulation. *Eur. J. Plant. Pathol.* 146 793–806. 10.1007/s10658-016-0957-z

[B65] Smyth R. A. Cifelli P. M. Ortori C. A. Righuetti K. Lewis S. Erskine P. (2010). Garlic as an inhibitor of *Pseudomonas aeruginosa* quorum sensing in cystic fibrosis—a pilot randomized controlled trial. *Pediatr. Pulmonol.* 45 356–362. 10.1002/ppul.21193 20306535

[B66] Soto-Aceves M. P. Cocotl-Yañez M. Merino E. Castillo-Juárez I. Cortés-López H. González-Pedrajo B. (2019). Inactivation of the quorum-sensing transcriptional regulators LasR or RhlR does not suppress the expression of virulence factors and the virulence of *Pseudomonas aeruginosa* PAO1. *Microbiology* 165 425–432. 10.1099/mic.0.000778 30707095

[B67] Stokes J. M. Yang K. Swanson K. Jin W. Cubillos-Ruiz A. Donghia N. M. (2020). A deep learning approach to antibiotic discovery. *Cell* 181 475–483. 10.1016/j.cell.2020.01.021 32302574

[B68] Tang H. Porras G. Brown M. M. Chassagne F. Lyles J. T. Bacsa J. (2020). Triterpenoid acids isolated from *Schinus terebinthifolia* fruits reduce *Staphylococcus aureus* virulence and abate dermonecrosis. *Sci. Rep.* 10:8046. 10.1038/s41598-020-65080-3 32415287 PMC7229044

[B69] Totsika M. (2016). Benefits and challenges of antivirulence antimicrobials at the dawn of the post-antibiotic era. *Drug Deliv. Lett.* 6 30–37. 10.2174/2210303106666160506120057

[B70] Townsley L. Shank E. A. (2017). Natural-product antibiotics: cues for modulating bacterial biofilm formation. *Trends Microbiol.* 25 1016–1026. 10.1016/j.tim.2017.06.003 28688575 PMC5701842

[B71] Turovskiy Y. Kashtanov D. Paskhover B. Chikindas M. L. (2007). Quorum sensing: fact, fiction, everything in between. *Adv. Appl. Microbiol.* 62 191–234. 10.1016/S0065-2164(07)62007-3 17869606 PMC2391307

[B72] Waheed H. Xiao Y. Hashmi I. Zhou Y. (2020). The selective pressure of quorum quenching on microbial communities in membrane bioreactors. *Chemosphere* 247:25953. 10.1016/j.chemosphere.2020.125953 32069724

[B73] Welsh M. A. Eibergen N. R. Moore J. D. Blackwell H. E. (2015). Small molecule disruption of quorum sensing cross-regulation in *Pseudomonas aeruginosa* causes major and unexpected alterations to virulence phenotypes. *J. Am. Chem. Soc.* 137 1510–1519. 10.1021/ja5110798 25574853 PMC4372995

[B74] Yao Z. Wang D. Wu X. Lin Z. Long Xi Liu Y. (2019). Hormetic mechanism of sulfonamides on *Aliivibrio fischeri* luminescence based on a bacterial cell-cell communication. *Chemosphere* 215 793–799. 10.1016/j.chemosphere.2018.10.045 30352376

[B75] Zakeri B. Lu T. K. (2013). Synthetic biology of antimicrobial discovery. *ACS Synth. Biol.* 2 358–372. 10.1021/sb300101g 23654251 PMC3716841

[B76] Zetterström E. C. Hasselgren J. Salin O. Davis R. A. Quinn R. J. Sudin C. (2013). The resveratrol tetramer (-)-hopeaphenol inhibits type III secretion in the Gram-negative pathogens *Yersinia pseudotuberculosis* and *Pseudomonas aeruginosa*. *PLoS One* 8:e81969. 10.1371/journal.pone.0081969 24324737 PMC3853165

[B77] Zhang Y. Sass A. Van Acker H. Wille J. Verhasselt B. Van Nieuwerburgh F. (2018). Coumarin reduces virulence and biofilm formation in *Pseudomonas aeruginosa* by affecting quorum sensing, type III secretion and C-di-GMP levels. *Front. Microbiol.* 9:1952. 10.3389/fmicb.2018.01952 30186266 PMC6110822

[B78] Zhao J. Chen M. Quan C. S. Fan S. D. (2015). Mechanisms of quorum sensing and strategies for quorum sensing disruption in aquaculture pathogens. *J. Fish Dis.* 38 771–786. 10.1111/jfd.12299 25219871

